# Radiological and Clinical Findings of Non-bifurcating Carotid Artery With Aberrant Course: A Case Report and Review of the Literature

**DOI:** 10.7759/cureus.25917

**Published:** 2022-06-13

**Authors:** Anas U Khan, Houman Sotoudeh, Siddhartha Gaddamanugu, Omid Shafaat, Celene Hadley, Aparna Singhal

**Affiliations:** 1 Medicine, University of Alabama at Birmingham, Birmingham, USA; 2 Radiology, University of Alabama at Birmingham, Birmingham, USA; 3 The Russell H. Morgan Department of Radiology and Radiological Science, Johns Hopkins University School of Medicine, Baltimore, USA; 4 Radiology, M & S Radiology Associates, San Antonio, USA

**Keywords:** vascular imaging, ct angiogram, anatomical variation, internal carotid artery (ica), non-bifurcating cervical carotid artery

## Abstract

A non-bifurcating carotid artery is a rare variation in the carotid circulation. Here we present a rare case of a non-bifurcating carotid artery with an aberrant course of the internal carotid artery incidentally discovered in a patient who presented to the trauma center after a fall. To our knowledge, this is the first reported case of a non-bifurcating carotid artery with an aberrant course of the internal carotid artery. The embryonic mechanisms of this variation and the available literature regarding this condition are also reviewed. Knowing this variation is necessary before considering vascular intervention of the neck and ear surgery to avoid vascular injury and complications.

## Introduction

The common carotid artery (CCA) normally divides into two branches: the internal carotid artery (ICA) and the external carotid artery (ECA). This division typically occurs at the C4 vertebra or thyroid cartilage level. In very rare cases, the CCA does not bifurcate and instead directly gives rise to the branches of the ECA and continues intracranially as the ICA. A retrospective analysis of MR angiograms revealed an incidence of 0.21% for this variation in a Japanese cohort (six cases) out of 2866 magnetic resonance angiography studies [1]. This same study found no preference for sex or laterality. In general, this condition is an incidental finding. However, a recently reported variation of the non-bifurcating carotid artery was associated with stroke in a young male patient [2]. There are less than 25 cases reported in the literature. So far, the co-existence of a lateralized ICA has not been reported in the literature to our knowledge. On imaging, lateralized ICA is recognized by the protrusion of ICA into the anterior middle ear and the genu of the ICA seen lateral to a line perpendicular to the mid-portion of the basal turn of the cochlea. Inferior tympanic canaliculus is normal, which helps to differentiate it from an aberrant ICA [3].

The carotid artery bifurcation vascular anomalies are very important to diagnose for the interventional and diagnostic radiologists as missed diagnosis could cause complications during vascular surgeries of the head and neck [4].

## Case presentation

**Figure 1:**
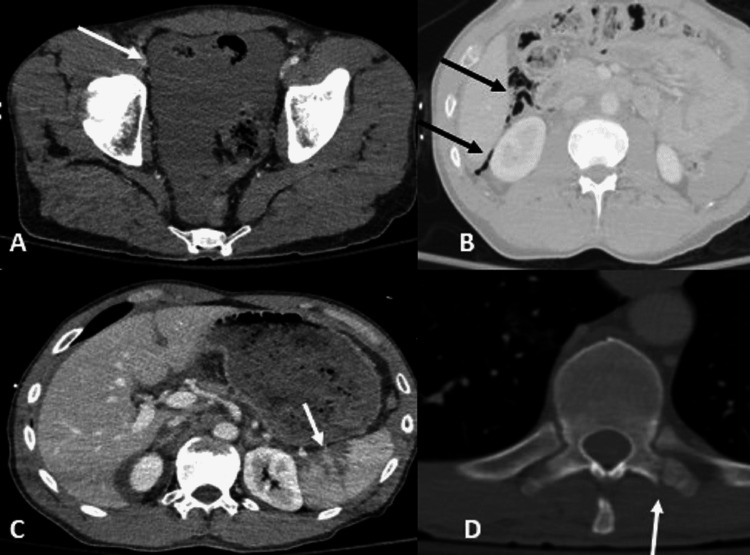
Postcontrast CT shows a large amount of free fluid in the abdomen and pelvis (A, white arrow), free air in right upper abdomen (B, black arrows), Splenic laceration (C, white arrow), and fracture of the left T10 transverse process (D, white arrow). A and C: CT with soft tissue window, B: CT with lung window, and D: CT with bone window.

A 30-year-old man with a history of polysubstance abuse and hepatitis C presented to the emergency room after falling from scaffolding 20 feet high with a loss of consciousness. Upon admission, the vital signs were normal (BP: 119/61 mmHg, HR: 72/min, RR: 22/min, and SpO2: 100%). The patient had thoracoabdominal injuries on trauma CTs of the chest and abdomen with large hemoperitoneum, extraluminal air in the right upper abdomen with multiple splenic lacerations, and fracture of the left T10 transverse process (Figure 1).

**Figure 2:**
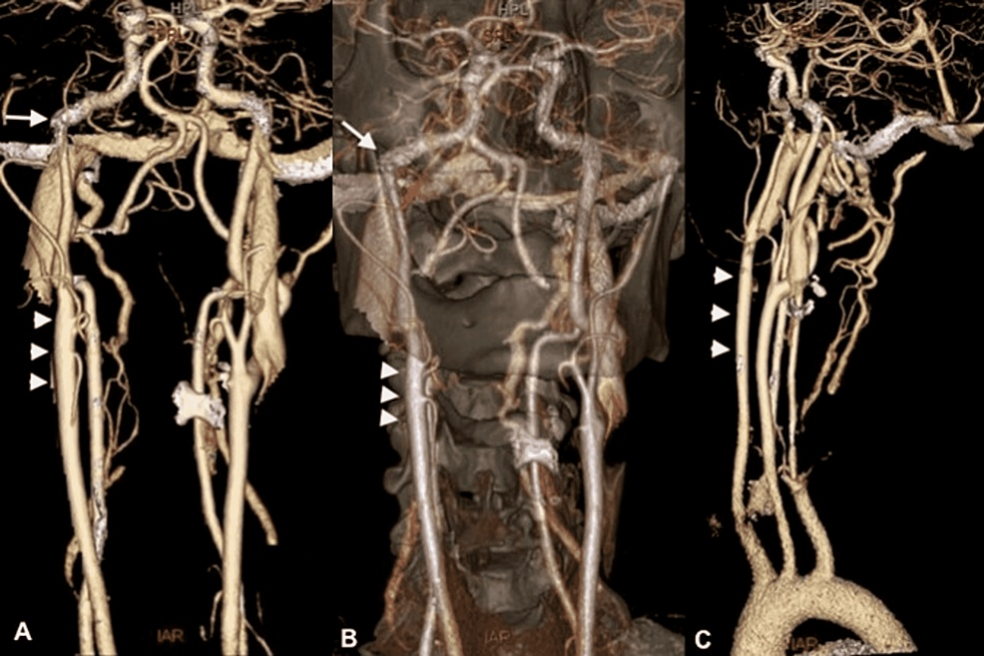
CTA neck shows a non-bifurcating carotid artery (NBCA) giving rise to the external carotid artery branches (A and B, arrow heads. The inferior branch: Superior Thyroid Artery. Two middle branches with close origin: Lingual and Facial Arteries. The superior branch: Superior Temporal Artery ), entering the skull base as the internal carotid artery (C, arrow heads), however with an additional variant, a lateralized carotid artery at the skull base (A, B, arrows).

**Figure 3:**
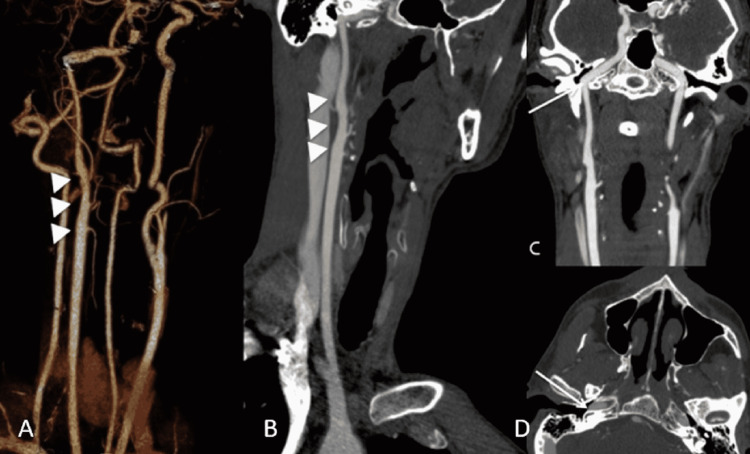
Oblique Volume rendered (A) and Maximum Intensity Projection (MIP) (B) images show the non-bifurcating carotid artery (NBCA) with external carotid artery branches (A, B, arrowheads. The inferior branch: Superior Thyroid Artery. Two middle branches with close origin: Lingual and Facial Arteries. The superior branch: Superior Temporal Artery). The Coronal MIP (C) and axial CTA (D) images further depict the lateralized course of the right internal carotid artery compared to the normal left side (arrows).

The neck CTA (Figures 2, 3) incidentally demonstrated a non-bifurcating right carotid with the right internal carotid as a single branch of the common carotid artery. In the absence of the right external carotid artery, the ECA branches arose from the upper portion of the right CCA. Also, an aberrant course of the right ICA was noted in the skull base with a lateral projection toward the right middle ear cavity. The patient was without neurologic symptoms. Subsequently, the patient underwent exploratory laparotomy, splenectomy (during laparotomy, the surgeon decided to perform splenectomy despite stable normal vital signs), and primary duodenal injury repair. The patient was ultimately discharged without any new neurological symptoms or other complications.

## Discussion

During embryogenesis, the CCA arises from the third aortic arch. The cervical ICA arises from the third aortic arch, and the intracranial ICA arises from the dorsal aorta. On the other hand, the ECA arises from the ventral aorta and the first two aortic arches. Deviation from this process can lead to the formation of such anomalies as a non-bifurcated carotid artery. Two main mechanisms have been proposed for how this occurs: 1) segmental agenesis of the ICA with anastomosis of the proximal ECA and distal ICA, and 2) agenesis of the ECA with its branches arising from the terminal segment of the CCA or proximal ICA [2,5-8]. A protrusion of the remnant ICA has been reported in a few cases, with one such case associated with a stroke [2,5]. In the absence of a stump at the expected bifurcation, anomalous development of the third aortic arch and proximal ICA agenesis with anastomosis of the proximal ECA and distal ICA are proposed, as would be postulated in our case [8]. There has been a recently proposed nomenclature for the subtype of cases with a stump to be called hypogenesis of the proximal ICA and those without the stump to be named agenesis of the proximal ICA [9].

The majority of these cases have been incidental except for a recently reported variation of the non-bifurcating carotid artery associated with a stroke. There have been cases of atherosclerosis and resultant stenosis associated with non-bifurcating carotid arteries (NBCAs), treated with carotid artery stenting or endarterectomy [5,6]. Our case demonstrated no atherosclerotic stenosis. Pathologic features of carotid plaques are similar in the case of an NBCA or normal bifurcation [5-7]. Additional coexisting pathologies reported include post-traumatic dissection, MCA stenosis, PCA occlusion, intracranial aneurysms, transverse sinus dural AV fistula, carotid-cavernous fistula, etc. [7-9]. In the study by Unchino et al., the facial-lingual (F-L) trunk, the ECA, the distal trunk, and the occipital artery (OA) were the most common branching order patterns from proximal to distal [1]. Lateralization of ICA is a developmental variation of unknown etiology, typically an incidental and isolated finding, although it has been implicated in causing tinnitus [10]. Coexistence with an NBCA has not been reported previously to our knowledge.

Clinical implications of NBCA are not necessarily known due to the low number of reported cases. Reported procedure-related implications include the difficulties with guidewire placement in the ECA during carotid angiography and stenting in the absence of ECA. Changing the interventional approach has been needed in such cases, as demonstrated by Sase et al. and Sasaki et al. [5,11]. Intra-arterial delivery of chemotherapy drugs or embolization devices can also be challenging in cases of the NBCA in head and neck cancers because of the connection between the branches of the ECA and ICA [4,12,13].

## Conclusions

We present a case of a very rare carotid anomaly in the form of a non-bifurcated carotid artery. While coexisting pathologies have been reported for this condition, concrete determination of clinical implications has not occurred, mainly due to the low number of reported cases. In our report, we demonstrate the presence of an additional anomaly in the form of a lateralized course of the ICA at the skull base, which to our knowledge has not been reported.
